# Extracellular enolase of *Candida albicans* is involved in colonization of mammalian intestinal epithelium

**DOI:** 10.3389/fcimb.2014.00066

**Published:** 2014-06-03

**Authors:** Richard C. Silva, Ana Carolina B. Padovan, Daniel C. Pimenta, Renata C. Ferreira, Claudio V. da Silva, Marcelo R. S. Briones

**Affiliations:** ^1^Departamento de Microbiologia, Imunologia e Parasitologia, Universidade Federal de São PauloSão Paulo, Brazil; ^2^Disciplina de Infectologia, DMED, Universidade Federal de São PauloSão Paulo, Brazil; ^3^Laboratório de Bioquímica e Biofísica, Instituto ButantãSão Paulo, Brazil; ^4^Universidade Federal de UberlândiaUberlândia, Brazil

**Keywords:** *Candida albicans*, enolase, gene sharing, cell adhesion, infection

## Abstract

Enolase is secreted by *Candida albicans* and is present in its biofilms although its extracellular function is unknown. Here we show that extracellular enolase mediates the colonization of small intestine mucosa by *C. albicans*. Assays using intestinal mucosa disks show that *C. albicans* adhesion is inhibited, in a dose dependent mode, either by pretreatment of intestinal epithelium mucosa disks with recombinant *C. albicans* enolase (70% at 0.5 mg/ml enolase) or by pretreatment of *C. albicans* yeasts with anti-enolase antibodies (48% with 20 μg antiserum). Also using flow cytometry, immunoblots of conditioned media and confocal microscopy we demonstrate that enolase is present in biofilms and that the extracellular enolase is not an artifact due to cell lysis, but must represent functional secretion of a stable form. This is the first direct evidence that *C. albicans'* extracellular enolase mediates colonization on its primary translocation site. Also, because enolase is encoded by a single locus in *C. albicans*, its dual role peptide, as glycolytic enzyme and extracellular peptide, is a remarkable example of gene sharing in fungi.

## Introduction

The fungus *Candida albicans* is a member of the microbiota of healthy individuals. However, in immunocompromised hosts it causes infections ranging from superficial mucosal to invasive systemic, usually fatal, manifestations (Odds et al., 1988; Dalle et al., 2010). The ability to adhere to surfaces is essential for these infections, because it enables the colonization and invasion of specific host niches. The gastrointestinal (GI) tract, although not exclusive, is the main *C. albicans* reservoir in humans, from which systemic infections are predominantly derived by translocation (Voss et al., 1994; Nucci and Anaissie, 2001). Specialized enterocytes (Goblet cells) secrete large amounts of mucus whose diversity and rapid turnover play an important role in limiting the multiplication of *Candida* cells on the gastrointestinal tract (Senet, 1997; Scott and Hancock, 2000). *C. albicans* secretory aspartyl proteinase, that degrades intestinal mucus, may contribute to pathogenicity by facilitating the penetration of the mucus barrier and the subsequent adhesion/invasion of epithelial cells (Colina et al., 1996). The efficiency of these adhesion/invasion steps is dependent on the expression of different *C. albicans* molecules that interact with different host receptors. A variety of adhesins are involved in binding to a wide array of proteins on the host's cell surfaces, including extracellular matrix components (ECM) such as laminin, fibronectin, and fibrinogen (Senet, 1997).

Enolase is one of the most abundantly expressed cytosolic enzymes in many organisms (Holland and Holland, 1978), and considered a multifunctional protein, because it performs different functions besides its primordial role in the glycolytic pathway (Pancholi, 2001). This is *gene sharing*, where an unduplicated gene acquires an additional function while maintaining its original one in the same organism (Tracy and Hedges, 2000). For example, enolase is also a heat shock protein involved in thermotolerance and growth control of *Saccharomyces cerevisiae* (Iida and Yahara, 1985) and a structural component of crystallins in birds, lampreys, fishes and reptiles (Piatigorsky, 2003). As observed in many other microorganisms and cells, *C. albicans* enolase can bind to plasminogen and plasmin. A possible mechanism for its increased ability to cross the human brain endothelial cells was proposed (Jong et al., 2003). Also, several studies have shown that enolase can mediate adhesion by interaction with extracellular matrix proteins such as fibronectin and laminin, although the underlying mechanisms are not fully understood (Carneiro et al., 2004; Esgleas et al., 2008; Castaldo et al., 2009; Donofrio et al., 2009). The enolase of *C. albicans* promotes a strong humoral immune response in patients with invasive candidiasis (Strockbine et al., 1984) and has been characterized as an important allergen in inhalant allergies to fungi (Ito et al., 1995). Also, it can be detected in culture medium and blood of patients (Sundstrom et al., 1994), being an important marker for invasive candidiasis (Walsh et al., 1991). It is also found on the cell surface (Eroles et al., 1997), in association with glucans in the cell wall (Angiolella et al., 1996).

Although previous studies indicate that *C. albicans* enolase is an immunodominant antigen (Angiolella et al., 1996), its precise role in *C. albicans* pathogenesis is still unknown. Here we show that pretreatment of GI epithelia with enolase blocks adhesion. Incubation of *C. albicans* with anti-enolase antibodies has a similar effect. We also demonstrate that enolase is secreted to the extracellular medium and biofilms. Our data also show that extracellular enolase derived from simple fungal lysis has a very short half life outside the cells suggesting that the proper extracellular form is not an artifact or an accidental leakage but it is actively secreted. Here we provide for the first time a potential role for the extracellular enolase in the gastrointestinal mucosae, the major translocation site of *C. albicans*. Our findings also suggest a novel example of gene sharing in fungi because enolase is encoded by a single locus in *C. albicans*. From our data we predict that enolase ligands might be present on the cell surface of the intestinal epithelia or in the thick and complex structure of mucus covering its surface.

## Materials and methods

### Microorganisms and growth conditions

Unless otherwise stated, *C. albicans* strain SC5314, kindly provided by Dr. A. Mitchell—Carnegie Mellon University, USA; *C. albicans* clinical isolate L296 (high biofilm-forming strain), kindly provided by Dr. Arnaldo L. Colombo—UNIFESP, LEMI, Brazil; *Saccharomyces cerevisiae* strain S288C, kindly provided by Dr. Beatriz A. Castilho—UNIFESP, Brazil and the reference *C. albicans* strain ATCC90029 (low biofilm-forming strain), were grown in YPD medium (1% yeast extract, 2% peptone, 2% dextrose) overnight with rotary agitation at 30°C. Cells were counted in a hemacytometer (Hirschmann EM Techcolor) and adjusted to the desired concentration immediately before the experiments. To produce hyphae, *C. albicans* yeasts were diluted to 1.0 × 10^7^ cells/ml in fetal bovine serum containing 5 mg/ml dextrose and incubated at 37°C with agitation for 3 h. *Escherichia coli* strain DH5-α was used for plasmid transformation. Strain BL21 (DE3) was used for recombinant protein expression. *E. coli* strains were grown overnight in LB medium supplemented with 100 μg/ml ampicilin or 15 μg/ml kanamicyn at 37°C with constant agitation.

### Expression and purification of recombinant enolase in *E. coli*

The complete coding region of *C. albicans* enolase gene was PCR amplified from genomic DNA with the following oligonucleotides: EnoexpresF:(5'-CGGGATCCATGTCTTACGCCACTAAAATC-3') and EnoexpresR: (5'-ATAGTTTAGCGGCCGCTTACAATTGAGAAGCCTTT-3'). The insert was subcloned between *Not* I and *Bam*HI restriction sites in plasmid pET-28a (+) (Novagen), in fusion with a Histidine tag at the N-terminal portion according to (Sambrook et al., 1989). This construction was transformed into *E. coli* BL21 (DE3). An overnight culture was inoculated into LB medium containing Kanamycin (15 μg/mL) and *C. albicans* enolase expression was then induced with 1 mM isopropyl-β-D-thiogalactopyranoside (IPTG) at 37°C for 2 h. After centrifugation, cells were lysed and the recombinant protein (His_6_-enolase) was purified under native conditions in pre-packed Ni-sepharose columns (Clontech) according to the manufacturer's instructions. The eluted protein was dialyzed for 36 h at 15°C against phosphate-buffered-saline (PBS) pH 7.6. Protein concentration was determined with the Bradford assay (Bradford, 1976) and by SDS-PAGE analyses. To assess the purity of the recombinant protein, samples were analyzed by Coomassie blue and silver nitrate staining SDS-polyacrylamide gel electrophoresis.

### Generation of polyclonal sera against his_6_-enolase

Purified His_6−_enolase was used to generate specific immune rabbit polyclonal sera. One hundred and fifty microgram of the protein with complete and incomplete Freund's adjuvant (Sigma) for the first and the other two doses respectively, at 20-days intervals, was subcutaneously injected in 2 adult female New Zealand white rabbits. To obtain the pre-immune sera, aliquots of blood were collected before the first immunization. Whole blood was collected 55 days after the first immunization. Sera were obtained by incubating the blood for 30 min at 37°C and overnight at 4°C, followed by centrifugation under 1000 rpm for 15 min at 4°C. All sera were stored at −80°C.

### Preparation of cell lysates

5 × 10^8^ yeasts were resuspended in lysis solution (20 mM Tris pH 8.0, 2 mM MgCl_2_, 2 mM EGTA and 150 mM NaCl) with protease inhibitors (1 mM phenylmethylsufonyl fluoride (PMSF), 2 mM benzamidin, 1 μg/ml leupeptin, 10 μg/ml pepstatin A, 4 μg/ml aprotinin, and 1 μg/ml antipain) and phosphatase inhibitors (10 mM Sodium Pyrophosphate and 1 mM Sodium Fluoride). Cell suspensions were vortexed and lysis was done by sequential freezing/thawing. After centrifugation at 13,000 rpm, for 15 min at 4°C, the supernatant (soluble fraction) was separated from the cellular debris and stored at −80°C prior to each use. For spent medium extraction, *C. albicans* SC5314 cultures in minimum and YPD media were grown at 37°C for 1–3 days. After incubation, 5 ml of media were separated from cells by centrifugation, filtered sterilized, concentrated 500× and frozen at −80°C. *E. coli* cell lysates of 10^7^ cells were prepared by sequential freezing/boiling procedures and the supernatant was separated from the cellular debris by centrifugation at 13,000 rpm for 5 min at 4°C, and stored at −80°C.

### Biofilm formation and protein matrix separation

*C. albicans* SC5314 biofilm was induced according to the protocol described elsewhere (Hawser and Douglas, 1994), with minor modifications. Biofilms were grown on 150 mm^2^ culture bottles, using minimal medium (0.67% YNB, Difco) supplemented with 50 mM glucose. Biofims were allowed to grow during 66 h, with medium replacement every 24 h. The spent medium was removed and biofilms were washed twice with 20 ml of cold PBS to remove non-adherent cells. 10 ml of cold PBS were added into each bottle and using cell scrappers, all biofilms and cells were detached from the bottles. The biofilm suspension was vigorously vortexed to disassemble cells from biofilm matrix, which were separated by centrifugation at 4000 rpm, 8 min, 4°C. Biofilm matrix supernatant was then filtered (0.22 μm filters, TPP), concentrated 500 times using Centricon Plus–20 (5000 mW—Millipore) and stored at −80°C.

### Western blot

Cell lysates and proteins were resuspended in 4 × Laemmli buffer, boiled for 5 min, separated by SDS-PAGE (Laemmli, 1970) and transferred to poly (vinylidene difluoride) membranes (GE Healthcare) (Towbin et al., 1979). Membranes were blocked overnight at 4°C with 5% non-fat dry milk in PBS-0.1% tween 20 and probed with: Pre-immune rabbit serum (1:500 in PBS); anti-His_6_-enolase (1:2000 in PBS); anti-His_6_-tag antibody—Santa Cruz Biotechnology (1:1000 in PBS). Membranes were then incubated with anti-rabbit IgG or anti-mouse IgG secondary antibodies (Sigma) at 1:1000 for 1 h in an orbital shaker. Detection was performed with the ECL-PLUS (GE Healthcare) according to the manufacturer's suggestions.

### Enolase decay assay

*C. albicans* SC5314 was grown in minimum medium at 37°C until the stationary phase. The culture was separated into two batches. The first batch was then separated again. One flask was maintained at room temperature and the other at 37°C without shaking for 5 h. The second batch was rapidly centrifuged to separate the cells from conditioned medium, filtered and then separated again. One flask was maintained at room temperature and the other at 37°C without shaking for 5 h. Aliquots of 5 ml were removed in different time points: 0 min, 30 min, 1, 2, 3, 4, and 5 h, concentrated 500 times (to 10 μ l) and stored at −80°C.

### Flow cytometry

*C. albicans* SC5314 was grown in YPD medium, overnight, at 37°C and 200 rpm. Two aliquots of 10^7^ yeasts were separated. The first aliquot was fixed in 3.5% formaldehyde for 1 h, washed in PBS and incubated overnight with anti His_6_-enolase (diluted 1:80 in PGN + 1% saponin) (permeabilized cells). The second aliquot was first incubated with anti His_6_-enolase without saponin and then was fixed (non-permeabilized). After consecutive washes in PBS, cells were incubated with anti-rabbit IgG marked with fluorescein (FITC) (diluted 1:100 in PBS) for one 1 h, at room temperature. After two washes, the number of yeasts was estimated with a fluorescent cytometer Becton-Dickinson, counting 10^4^ cells. As a control, permeabilized and non-permeabilized cells were incubated with pre-immune rabbit serum. For the enolase uptake assay, a culture of *C. albicans* SC5314 was processed as described above. Five aliquots of 2 × 10^4^ cells were separated. The first aliquot was incubated overnight at 4°C with the elution buffer of the recombinant protein purification and the other four aliquots were incubated with different concentrations of His_6_-enolase: 1.25, 2.5, 5, and 10 μg. After washes with PBS, cells were fixed with 3.5% paraformaldehyde for 1 h. Then, cells were incubated with anti-His_6_ (diluted 1:20 in PGN) for 1 h, washed and incubated with anti-rabbit-IgG labeled with FITC (diluted 1:50 in PBS). As control, the recombinant enolase was coupled to latex beads (100 μ g of His_6_-enolase to 10^8^ beads) and incubated in coupling buffer (200 mM sodium carbonate, 500 mM sodium chloride, pH 8.5) overnight, at 37°C, with agitation. Beads were subsequently washed and blocked with BSA solution in PBS (20 mg/ml) and then incubated with anti-His_6_ (diluted 1:20 in PGN) for 1 h. After this period, they were washed and incubated with anti-mouse IgG labeled with FITC (diluted 1:50 in PGN). After two washes cells were analyzed in a Becton-Dickinson flow cytometer (10,000 events).

### Immunofluorescence microscopy

*C. albicans* were centrifuged, washed with PBS and fixed with 3.5% formaldehyde in PBS for 1 h. After incubation, cells were washed at least five times with PBS and then 10 μ l were dripped on to glass slides to dry at room temperature. For biofilm formation, glass slides were coated with poly-lysine (10 mg/ml) (Sigma), dried and the biofilm was formed according to the protocol previously described. Slides containing yeasts, hyphae and biofilm were permeabilized with 1% saponin (BDH, Amersham, UK) in PGN (0.2% gelatin, 0.1% NaN_3_ in PBS) or incubated only with PGN for blocking (non-permeabilized cells) for 15 min. The coverslips were incubated with anti-His_6_-enolase (diluted 1:80 in PGN or PGN + saponin) overnight at 4°C and washed with PBS. They were subsequently incubated with anti-rabbit-IgG conjugated with Cy3 (Sigma) diluted 1:100 in calcofluor white (10 mg/ml) for 1 h, in the dark. After washing with PBS, coverslips were mounted in glycerol buffered with 100 mM Tris pH 8.6 and 0.1% p-phenylenediamine. Slides were examined under a confocal microscope (BioRad 1024-UV) coupled to a Zeiss Axiovert 100 microscope. Alternatively, images were acquired in an epifluorescence microscope.

### Adhesion assays

The adhesion assays were based on a modification of the protocol previously described by (Segal and Sandovskylosica, 1995). Five week-old male Balb/c mice were purchased from University of São Paulo - USP, Brazil. Experiments were performed in accordance with guidelines for animal use and care of the Federal University of São Paulo—UNIFESP and approved by the Ethics Committee in Research. Mice were maintained in pathogen free conditions and aged 8–12 weeks for the experiments. Mouse integral intestine was aseptically removed from sacrificed animals and a cut of approximately 0.4 inches was done after the end of the stomach and before the caecum. The small intestine (duodenum, jejunum, and ileum) was cut longitudinally, rinsed once in sterile PBS to remove aggregates and opened on a flat surface. Mice intestinal tissue disks (6 mm in diameter) were obtained with a sterile metal punch. Inhibition of *C. albicans* adhesion to mouse intestinal disks coated with recombinant enolase was performed as follows: disks were added to eppendorf tubes containing increasing concentrations of His_6_-enolase to a final volume of 1 ml in PBS. These tubes were then incubated for 2 h at 37°C in an orbital shaker. Disks were rinsed twice in PBS, added to eppendorf tubes containing 2.5 × 10^6^ *C. albicans* in PBS and incubated for 2 h at 37°C. Disks were rinsed 5 times to remove non adherent cells, homogenized and eluted in 1 ml of PBS. Pre-coated *C. albicans* adhesion assays were performed by preincubating 5.2 × 10^6^ *C. albicans* yeasts with different concentrations of purified anti His_6_-enolase for 2 h at 37°C. Cells were centrifuged at 3000 g for 3 min at 4°C, washed 3 times and eluted. Disks were added to cell suspensions and processed as previously described. All elutions were serially diluted and plated on YPD medium containing 34 μg of chloramphenicol/ml. Plates were incubated for 48 h at 30°C, and the number of attached microorganisms per tissue disk was estimated by counting *C. albicans* colony forming units (cfu).

## Results

### Potential role of biofilm enolase

**Figure 1:**
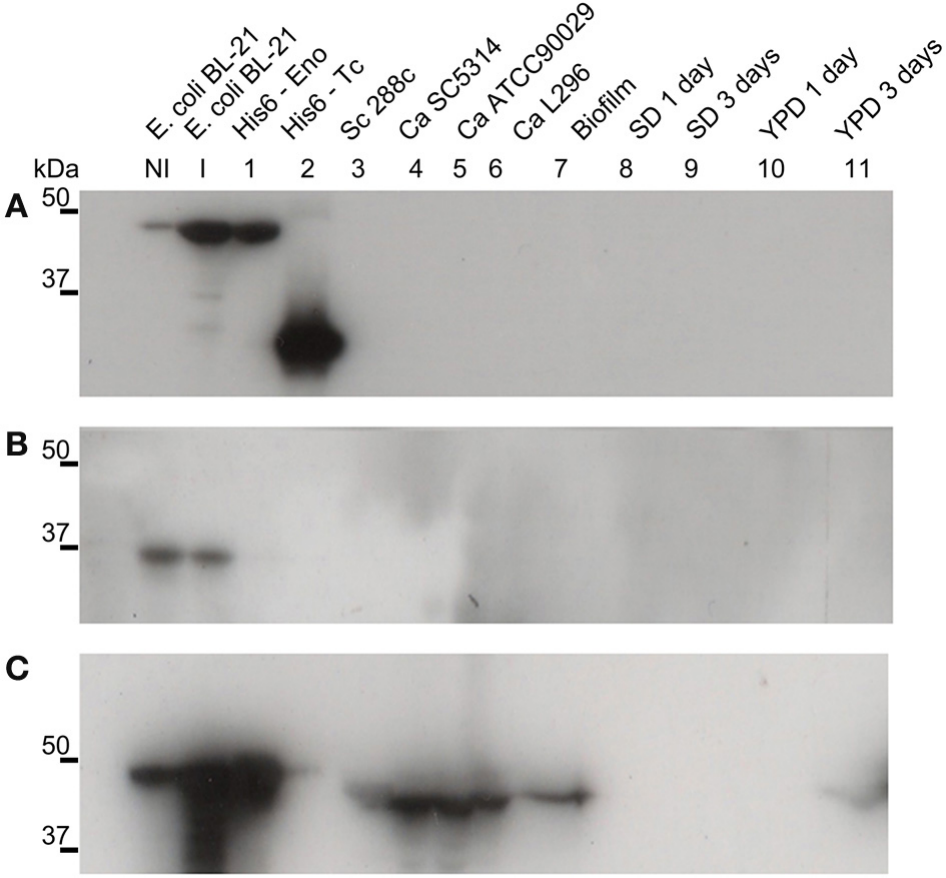
**Native enolase is recognized in *C. albicans* cell lysates, biofilm and supernatant of conditioned rich medium**. Western blots of enolase in biofilm and cell lysates of different *C. albicans* strains, according to: NI, non-induced *E. coli* BL-21 lysate; I, *E. coli* BL-21 expression induced for 2 h at 37°C lysate; 1, recombinant His_6_-enolase; 2, His_6_-*T. cruzi* 30 kDa protein used as control; 3, *S. cerevisiae* 288c cell lysate; 4, *C. albicans* SC5314 cell lysate; 5, *C. albicans* ATCC90029 cell lysate, 6, *C. albicans* L296 cell lysate; 7, *C. albicans* SC5314 biofilm matrix concentrated 500 times; *C. albicans* SC5314-conditioned medium are respectively: 8, minimal medium with 24 h growth; 9, minimal medium with 72 h growth; 10, YPD with 24 h growth; and 11, YPD with 72 h growth. **(A)** anti-His_6_ antibody (dilution 1:1000), shows the recognition of the recombinant protein of His_6_-*T. cruzi* and the His_6_-enolase, confirming its proper purification; **(B)** pre-immune rabbit serum (1:500), showing no recognition of fungal proteins and recognition of a non-specific low molecular weight band (below 37 kDa) within the *E. coli* extracts; and **(C)** anti His_6_-enolase (1:2000), showing recognition of the recombinant enolase and native protein in biofilms, cell lysates and spent medium.

The intracellular role of enolase as a glycolytic pathway enzyme is widely known. To investigate its potential extracellular role, suggested by its presence in the biofilm matrix, we produced a recombinant *C. albicans* enolase (Figure 1) and an anti-His_6_-enolase polyclonal rabbit serum. We found that native enolase can be detected in the biofilm matrix of *C. albicans* SC5314 and in cell lysates of three different *C. albicans* strains including the SC5314, one clinical isolate identified as a high biofilm producer (L296) and a reference strain that is a low biofilm producer (ATCC90029), used as control (Padovan et al., 2009) (Figure 1C). Only one band was observed in the biofilm matrix and also the homologous enolase in the cell lysate of *S. cerevisiae* S288C. A weaker signal was observed after 3 days in YPD medium (Figure 1C).

### Enolase is present on the cell surface

**Figure 2:**
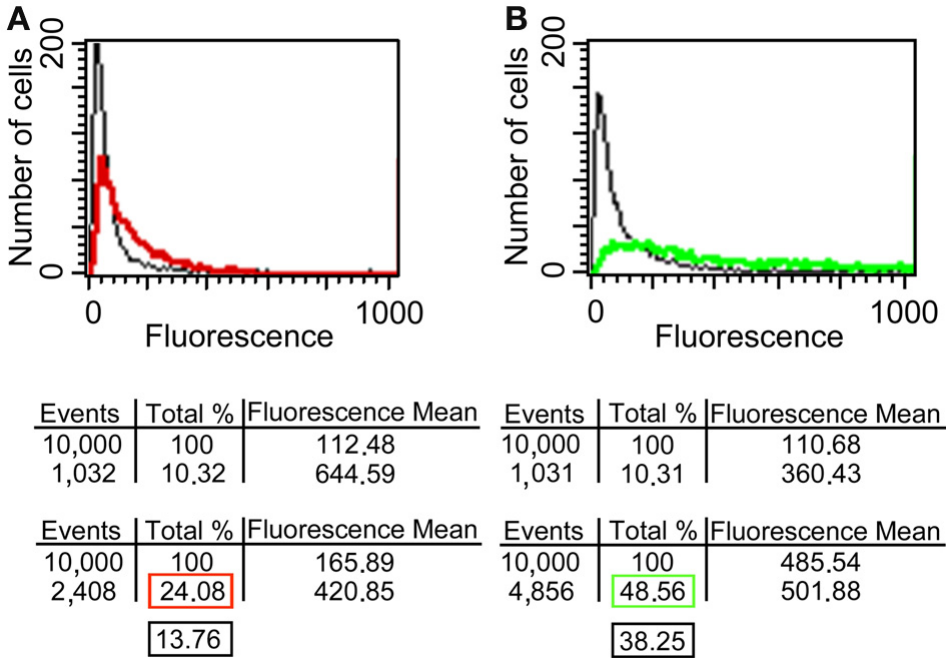
**Flow cytometry of enolase detected on yeast surface and in the whole cell. (A)** *C. albicans* SC5314 fixed and non-permeabilized cells were incubated with anti-His_6_-Enolase polyclonal serum (1:80), present slightly higher fluorescence (red line) than the relative fluorescence background of the untreated control cells (black line). **(B)** *C. albicans* SC5314 fixed and permeabilized cells with saponin 1% exhibit higher fluorescence (green line) compared with non-permeabilized cells. 10^4^ cells were analyzed per experiment. On average, 10% of them exhibited autofluorescence that were discounted of the experimental measurements highlighted in the red (non-permeabilized cells) and green (permeabilized cells) boxes in the final analysis. Black boxes depict the subtraction of fluorescence by autofluorescence values (Approximately 14 and 40% for non-permeabiled and permeabilized cells, respectively).

**Figure 3:**
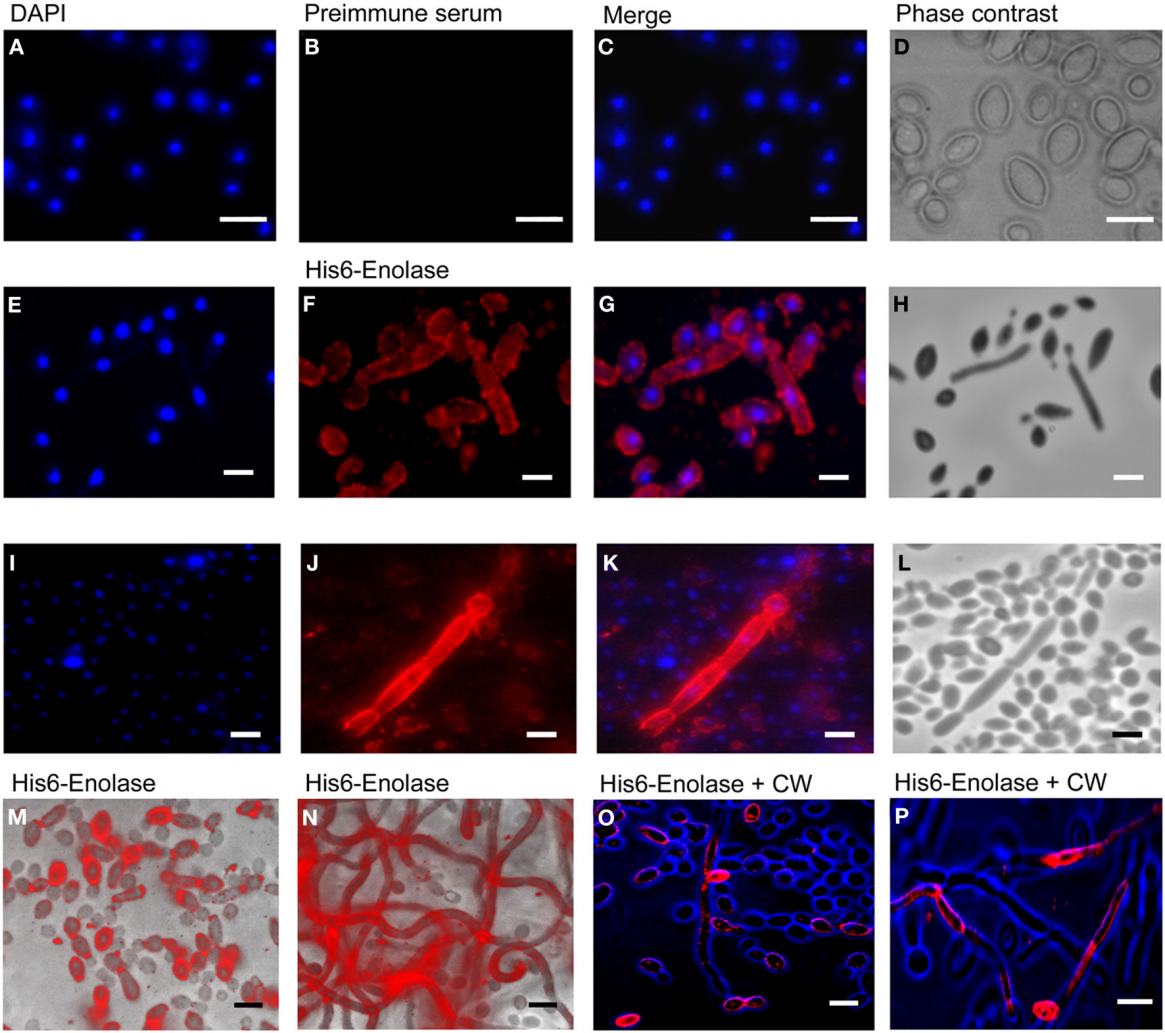
**Immunofluorescence of native enolase detected in yeasts, hyphae and biofilm-forming cells of *C. albicans* SC5314**. Immunofluorescence of yeasts permeabilized with saponin 1%: **(A)** DAPI (1:2000); **(B)** Pre-immune serum (1:80); **(C)** Merged images and **(D)** Phase contrast image. Immunofluorescence of non-permeabilized yeast and filaments cells: **(E)** DAPI (1:2000); **(F)** anti His_6_-enolase polyclonal serum (1:80); **(G)** Merged images and **(H)** Phase contrast image. Filaments permeabilized with saponin 1%: **(I)** DAPI (1:2000); **(J)** anti His_6_-enolase polyclonal serum (1:80); **(K)** Merged images and **(L)** Phase contrast image. Presence of enolase in biofilm cells permeabilized with saponin 1% and incubated with anti His_6_-enolase polyclonal serum (1:80): **(M)** immunofluorescence microscopy overlaid with phase contrast of the bottom part of the biofilm enriched with yeasts; **(N)** immunofluorescence microscopy overlaid with phase contrast of the upper part of the biofilm enriched with hyphae cells. Immunofluorescence microscopy images merge of enolase in non permeabilized biofilm cells stained with calcofluor white (CW) (1:200) and incubated with anti His_6_-enolase polyclonal serum (1:80): **(O)** bottom part of the biofilm with budding yeasts highly covered with enolase; **(P)** upper part of the biofilm enriched with hyphae. Bars: 10 μm.

Since it has been reported that enolase is present on the cell surface of yeasts (Pancholi, 2001), we tested the ability of the anti His_6_-enolase polyclonal rabbit serum to detect cell surface enolase in biofilm-forming cells. Flow cytometry data (Figure 2A) showed that enolase could be detected on the surface of 14% non-permeabilized yeasts cells, while in permeabilized cells; enolase was detected in 40% (Figure 2B). The growth conditions of cells used in this experiment (see Flow cytometry) might have induced the formation of germ-tubes and pseudo-hyphae, possibly explaining the flatline shift in fluorescence observed in Figures 2A,B. To verify whether cells in the biofilm also had enolase on their surface, immunofluorescence microscopy images of yeasts and hyphae were compared with biofilms (Figure 3). Figures 3A–D show that enolase was not recognized by the pre-immune serum. Figures 3E–H show enolase covering the cell surface of non-permeabilized yeasts. Diffused and stronger labeling was observed in permeabilized cells (Figure 3K) although several cells were not labeled. Figures 3M,N showed strong enolase signal in permeabilized biofilm-forming cells, both in yeasts attached to the slide, at the basal layer, as in hyphae and buds at the upper part of the biofilm. Non-permeabilized biofilm-forming cells exhibited less enolase signal on their surface (Figures 3O,P). Strikingly, in non-permeabilized biofilm-forming cells, the co-localization of enolase with calcofluor occurred in a few cells. These data support previous findings that enolase is present on the cell surface of *C. albicans* yeasts and indicate for the first time that enolase is also present on the surface of hyphae and biofilm-forming cells (yeasts and hyphae).

### Biofilm enolase is not derived from leakage of dead cells

**Figure 4:**
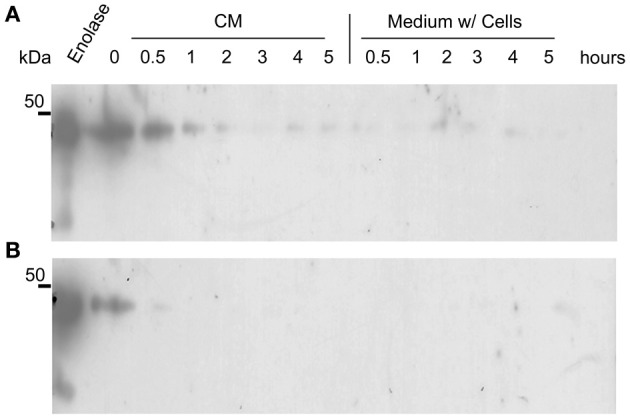
**Western blots of the kinetics of *C. albicans* SC5314 enolase decay in conditioned media**. Aliquots of 5 ml conditioned minimal medium (CM) only and stationary cultures (w/ = with cells) were maintained at room temperature **(A)** and at 37°C **(B)**, without agitation. Samples were removed and analyzed by western blot (Probed with anti His_6_-enolase). Numbers indicate the time interval of sampling in hours.

It could be argued that the presence of enolase in the biofilm matrix is a consequence of cytoplasm leakage of dead cells at the bottom layers of the biofilm, and therefore there would be no specific function for enolase in the biofilm matrix. To test whether native enolase could be detected in stationary cultures, *C. albicans* SC5314 was grown in minimal medium until reaching the stationary phase and aliquots of the culture medium with and without cells were taken from 0.5, 1, 2, 3, 4, and 5 h to check for the presence of native enolase. A decay in native enolase levels was observed at room temperature, with or without cells (Figure 4A), while a much faster decay was observed at 37°C at 30 min, especially in the cell-free conditioned medium (Figure 4B). To test whether cytosolic proteins could be detected in the biofilm due to cell lysis, we checked for the presence of Sui2p (eIF2α—The alpha subunit of the eukaryotic translation initiation factor 2), a cytoplasmic protein of *S. cerevisiae.* The *S. cerevisiae* Sui2p has 84% of similarity and 70% identity with its *C. albicans* homolog, CaSui2p (orf19.6213). We detected Sui2p and CaSui2p in cell extracts of *S. cerevisiae* and *C. albicans*, respectively, while in conditioned media it was weakly detected and not detected at all in the biofilm matrix (Data not shown).

### Short half-life of dead cell leaked enolase is not explained by reuptake

**Figure 5:**
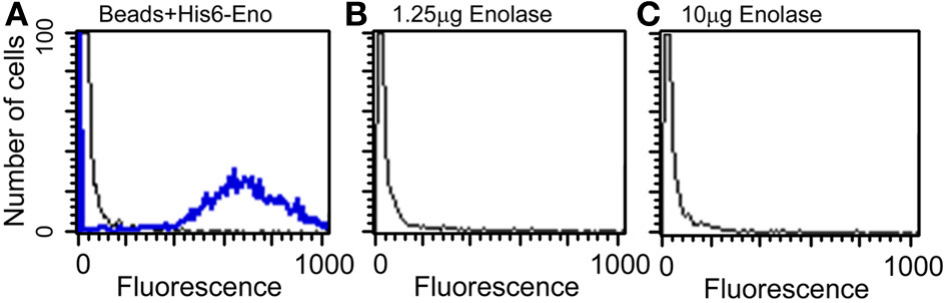
**Flow cytometry of *C. albicans* SC5314 incubated with purified enolase (enolase uptake assay). (A)** Control beads coated with His_6_-enolase were incubated with anti-His_6_-tag (1:80) (blue line) compared with *C. albicans* SC5314 incubated with the elution buffer of His_6_-enolase (black line). **(B)** Yeasts incubated with 1.25 μg of His_6_-enolase and subsequently incubated with anti-His_6_-tag (1:80) (no signal). **(C)** Yeasts incubated with 10 μg of His_6_-enolase and incubated as **(B)** (no signal).

Because enolase was detected on the cell surface of planktonic yeasts and hyphae and on biofilm forming cells, the enolase decay could also be explained by the enolase uptake from the external medium to the cell wall. To test this hypothesis, cultures of *C. albicans* SC5314 were incubated with 4 different concentrations of His_6_-enolase and its presence on the cell surface of yeasts was assessed by flow cytometry. Figure 5 shows that there was no recognition of the recombinant enolase on the surface of *C. albicans* cells in all the concentrations tested (1.25–10 μ g), as opposed to His_6_-enolase coated beads. These data suggest that enolase present on the cell surface is not a product of protein uptake from cell debris lysis.

Taken together the results above suggest that enolase in the biofilm matrix is not a product of cell lysis because it would be rapidly degraded at 37°C as occurred in yeast cell cultures (Figure 4B). Therefore enolase might be functionally secreted to the extracellular environment such as biofilms and to the cell surface where it is stable. These results also indicate that the presence of enolase on the cell surface is not a result of protein uptake from cell debris lysis.

### Blocking of cell surface enolase with anti-enolase inhibits *C. albicans* adhesion to mice GI tissue disks

**Figure 6:**
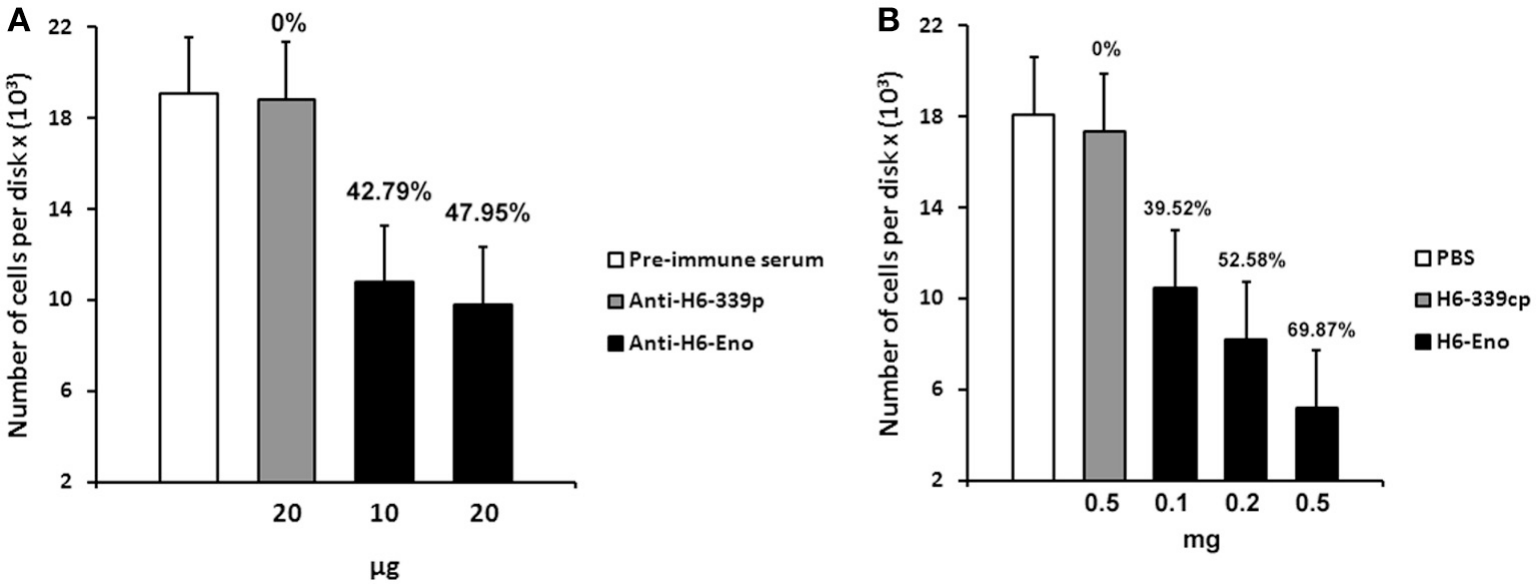
**Inhibition of *C. albicans* adhesion to mice gastrointestinal (GI) tissue disks. (A)** Preincubation of *C. albicans* yeasts strain SC5314 with different concentrations of purified polyclonal anti His_6_-enolase (10 and 20 μg) inhibited its adhesion to GI disks in comparison with the negative controls (20 μg of polyclonal purified anti His_6_-CaYlr339cp or pre-immune serum). Data represent mean number of cells per tissue disks × (10^3^) based on colony-forming units (cfu) counting of three independent experiments performed in triplicate. Bars represent the standard error (SE) of the means. Percentage values above the bars represent the percentage inhibition of adhesion with the negative control (anti His_6_-CaYlr339cp) considered as 0%. **(B)** Pretreatment of disks with increasing concentrations of the recombinant protein His_6_-enolase (0.1–0.5 mg) inhibited *C. albicans* strain SC5314 adhesion in a dose dependent manner in comparison with the negative controls (disk incubated with 0.5 mg of His_6_-CaYlr339cp in PBS or PBS alone). Data represent mean number of cells per tissue disks × (10^3^) based on colony-forming units (cfu) counting of three independent experiments performed in triplicate. Bars represent the standard error (SE) of the means. Percentage values above the bars represent the percentage inhibition of adhesion with the negative control (His_6_-CaYlr339cp) considered as 0%.

To investigate the role of cell surface enolase in adhesion, we first checked whether *C. albicans* yeasts preferentially associates with specific parts of mice small intestine *in vitro*. There was no difference in the number of yeasts adhering to disks obtained from three equal portions (proximal, medial and distal third parts) of small intestine tissues (Figure S1 supplemental). Therefore, we employed disks prepared from the whole small intestine in all our assays. *C. albicans* yeasts were pre-incubated with different concentrations of affinity-purified rabbit anti-serum and then incubated with mice GI tissue disks. There was no difference in the number of yeasts per tissue disks when *C. albicans* was blocked with the pre-immune serum or with the anti His_6_-CaYlr39cp (an antibody against the intracellular protein CaYlr339cp), used as a negative control. On the other hand, blocking *C. albicans* with 10 and 20 μg of anti His_6_-enolase decreased its adhesion to mice GI tissue disks to approximately 42 and 47 % respectively, in relation to anti His_6_-CaYlr339cp (Figure 6A). The inhibition of adhesion seemed to saturate when 10 μg of antibodies were used since twice of its concentration did not decrease proportionately the adhesion of yeasts to GI disks. In an attempt to verify if the decrease in adhesion observed was not due to the potential recognition and blockage of surface proteins rich in histidine regions in *C. albicans*, yeasts were also blocked with a monoclonal anti-His_6_-Tag antibody. There was no difference in adhesion in relation to the negative control (Data not shown). Also, viability assays indicated that this inhibition was not dependent on potential candidacidal activities promoted by the polyclonal antiserum raised against recombinant enolase (Figure S2 supplemental). These results indicate that the blockage of enolase present on the cell surface of *C. albicans* with specific antibodies against enolase impairs its adhesion to mice GI disks *in vitro*.

### Inhibition of *C. albicans* adhesion to mice GI tissue disks by purified recombinant enolase

Since the coating of cell surface enolase with antibodies was able to inhibit *C. albicans* yeasts adhesion to GI disks *in vitro*, we hypothesized that blocking potential enolase receptors on disks might also have an effect on *C. albicans* adhesion. To test this hypothesis, mice GI tissue disks were pre-incubated with different concentrations of recombinant His_6_-enolase. After incubation, disks were washed and then incubated with *C. albicans* yeasts. Strikingly, the incubation of disks with 0.5 mg of soluble purified His_6_-enolase inhibited *C. albicans* adhesion to GI disks at 70% in comparison to the purified protein His_6_-CaYlr339cp, used as a negative control. The decreased adhesion was dose dependent because incubation of disks with 0.1 and 0.2 mg of enolase inhibited adhesion in 39 and 52 % respectively (Figure 6B). There was no difference in the number of attached *C. albicans* cells per disk in relation to disks incubated with His_6_-CaYlr339cp in PBS or PBS alone. Together, these results indicate that recombinant enolase may be blocking specific host enolase receptor(s) on mice GI disks, thus inhibiting the adhesion of *C. albicans.*

## Discussion

We identified enolase in *C. albicans* biofilms by mass spectrometry of SDS-PAGE bands in an initial attempt to study biofilm composition (data not shown). Using western blot and confocal microscopy we confirmed this observation and also identified for the first time enolase in biofilm forming cells, buds and growing hyphae of *C. albicans* (Figures 1–3). In its cytosolic form, enolase is a protein of approximately 48 kDa, which catalyzes the dehydration of 2-phosphoglycerate to generate phosphoenolpyruvate in the glycolytic pathway and the reverse reaction in gluconeogenesis (Sundstrom and Aliaga, 1992) but its function in the biofilm, whether structural or regulatory, is still unknown. Our results confirmed previous studies demonstrating enolase in biofilms (Thomas et al., 2006) and in planctonic cells (Angiolella et al., 1996), and provided for the first time evidence of extracellular enolase in biofilm forming cells and hyphae. Interestingly, enolase seems to migrate with a slightly larger molecular weight in the biofilm matix relative to the enolase detected in whole cell extracts of *C. albicans*, suggesting that enolase is being post-translationally modified in the biofilm. Further studies are necessary to address this question. Our FACS analysis and immunoblots of conditioned media suggested that enolase presence within the extracellular milieu is probably functional and not artifactual, e.g., by lysis of dead cells (Figures 2, 4, 5). To address the function of the extracellular enolase we devised adhesion assays using yeasts coated with anti-enolase antibodies and mice GI disks coated with purified enolase. Our results clearly show an important effect of inhibition in both types of experiments. These assays are a modification of the assay described by Segal and Sandovskylosica (1995), and was carried out using mice small intestine tissue disks, comprising the duodenum, jejunum and ileum. Because no differences in adhesion were observed among three equal portions of the small intestine (Figure S1 supplemental), the whole small intestine portion was used in all experiments herein. These assays allowed us to demonstrate how preincubation of disks with enolase and of *C. albicans* with anti-enolase antibodies block the adhesion. The intestinal serosa comprise a large portion of the intestinal disks, therefore it could be argued that *C. albicans* would also adhere to this portion. However, it was previously demonstrated that yeasts preferentially attach to the mucosa and only a limited number of organisms attach to the serosa, making this technique a much more realistic *in vitro* model to the actual *in vivo* phenomenon of attachment (Sandovskylosica and Segal, 1989).

Inhibition of *C. albicans* adhesion is one of the most well documented activities mediated by antibodies, and distinct levels of inhibitions toward many cell wall antigens have already been described (Cotter et al., 1998; Laforce-Nesbitt et al., 2008). In this work, we demonstrated a role for cell surface enolase in adhesion by blocking *C. albicans* with a specific anti-enolase antibody. We found that neutralization of cell surface enolase inhibits its adhesion to mice GI tissue disks. The saturation of inhibition observed indicates that multiple *C. albicans* molecules mediate the adhesion to gastrointestinal disks *in vitro*. Our experiments also show that these results were not biased by possible direct candidacidal activity of antibodies (Figure S2 supplemental) or by the induction of germ tubes formation by antiserum or by contact with tissue disks *in vitro* (data not shown). The decrease in adhesion by anti-enolase antibodies is not without precedent. Blocking of cell surface enolase from *Paracoccidioides brasiliensis* (Donofrio et al., 2009), and *Streptococcus suis* (Esgleas et al., 2008) inhibited their adhesion to pulmonary epithelial and brain microvascular endothelial cells respectively.

To further characterize the role of enolase on adhesion, mice GI tissue disks were incubated with recombinant enolase in an attempt to mask possible receptor binding sites on host tissues. Pretreatment of GI disks strongly inhibited *C. albicans* adhesion in a dose-dependent manner (Figure 6B). Incubation of *C. albicans* with the recombinant enolase indicated that the inhibition observed is not a consequence of possible cytotoxic contaminants on the purified protein (data not shown). An important inhibition of *C. albicans* adhesion was also observed by Segal and Savage after pretreatment of mice duodenal disks with chitin soluble extracts (Segal and Savage, 1986). Our data are in line with a previous study reporting a decrease in adhesion of *S. suis*, serotype two, to epithelial cells exposed to recombinant enolase (Zhang et al., 2009). Altogether these results suggest that enolase directly interacted to specific epithelial receptors on mice intestinal disks, thus inhibiting *C. albicans* adhesion.

The intestinal mucosa comprises an epithelial layer arranged in individual columns. It is composed of two fractions: a loosely adherent layer easily removable by suction and a layer firmly attached to the mucosa, which actually constitutes the intestinal epithelial cells glycocalyx (Hecht, 1999; Scott and Hancock, 2000; Atuma et al., 2001). *C. albicans* can associate directly to epithelium cells or to the mucus layer covering these cells (Kennedy et al., 1987). In view of this fact and the report showing the association of *Streptococcus mutans* enolase to a highly glycoysylated oral mucin *in vitro* (Ge et al., 2004), it is possible to speculate that enolase could be binding to glycoproteins covering the epithelium or even to loosely adherent mucus, therefore inhibiting *C. albicans* adhesion. However, we do not exclude the possibility that enolase might have inhibited the adhesion by modifying mucosal properties or host receptors (Freter and Jones, 1983), rendering *Candida* cells unable to bind to intestinal disks. Further research is required to determine the specific host receptor(s) involved in such interaction. Since the adherence of *C. albicans* to different surfaces is paramount for biofilm formation, it is possible that enolase expressed on the surface of biofilm-forming cells could also contribute to the adhesion of *C. albicans* to different substrates with potential implications for biofilm adhesion and formation. Further experiments are required to test this hypothesis. Nevertheless, based on our data, we propose that the *C. albicans* enolase present on the cell surface is functional and does not represent an artifact. We also propose that surface enolase plays an important role in adhesion of *C. albicans* in its major translocation site. Also, because enolase is encoded by a single gene in *C. albicans* genome, its dual role as a glycolytic enzyme and as an extracellular component, exemplifies an interesting and novel case of gene sharing in fungi.

## Author contributions

Richard C. Silva, Ana Carolina B. Padovan, and Renata C. Ferreira conceived, performed and analyzed the data. Richard C. Silva, Ana Carolina B. Padovan and Marcelo R. S. Briones wrote the paper. Daniel C. Pimenta performed the preliminary mass spectra analysis of biofilms. Claudio V. da Silva helped to perform the immunofluorescence microscopy and flow cytometry experiments. Marcelo R. S. Briones conceived the experiments and supervised first authors. All authors analyzed the data and revised the final manuscript.

### Conflict of interest statement

The authors declare that the research was conducted in the absence of any commercial or financial relationships that could be construed as a potential conflict of interest.
