# Supplementary material for: Extracellular enolase of Candida albicans is involved in colonization of mammalian intestinal epithelium
Source: Front Cell Infect Microbiol. 2014 Jun 3;4:66. doi: 10.3389/fcimb.2014.00066 (PMC4042164; doi:10.3389/fcimb.2014.00066)
Supplement: Supplementary file 1 [file DataSheet1.PDF]

(Figure S1 - supplemental) Intestinal epithelial disk adhesion assay on proximal, medial and distal third parts of mouse small intestine. To compare the adhesion of C. albicans to different portions of small intestine, $2.5x10^6$ *C albicans* yeasts *were incubated with* mice small intestine cut in tree equal portions and processed as described in material and methods (item 2.10). There was no difference in the number of yeasts adhering to disks obtained from different intestine portions.

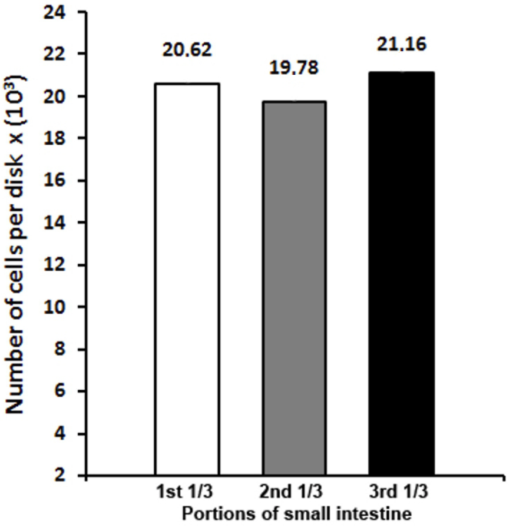

(Figure S2 - supplemental) Antisera raised against enolase do not present candidacidal activity

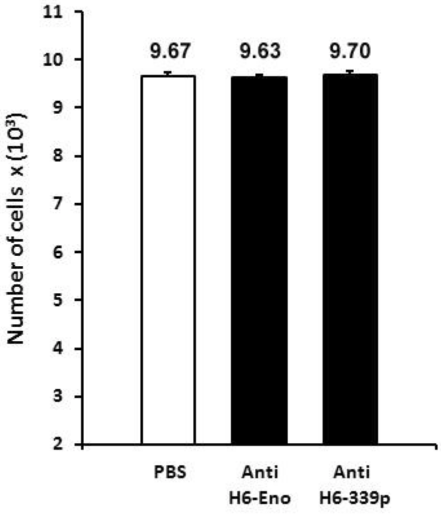

The candidacidal activity of anti His_6-enolase (purified from rabbit serum) was evaluated as previously described by Magliani et al. (1997), with some modifications. Briefly, 125 µl of a PBS suspension containing 5.2 x 10^4 yeasts were added to tubes containing 50 ng of the purified anti-His_6 serum to be tested. Cell suspensions were incubated at 37ºC for 2 h in an orbital shaker. All suspensions were then centrifuged at 3,000 g for 3 minutes at 4ºC, washed three times and eluted in 1 ml of PBS. All elutions were plated on YPD solid media containing chloramphenicol (34 µg/ml). Plates were incubated at 30ºC for 48 h for *C. albicans* colony forming units (cfu) determination. The number of cells obtained after the incubation of *C. albicans* with anti His_6-enolase did not differ in relation to cells incubated with the anti His_6-Caylr339cp in PBS or with PBS alone (negative control). Data represent means from three independent experiments performed in triplicate. Bars represent the standard error (SE).

Magliani W, Conti S, de Bernardis F, Gerloni M, Bertolotti D, Mozzoni P, Cassone A and Polonelli L (1997) Therapeutic potential of antiidiotypic single chain antibodies with yeast killer toxin activity. *Nat. Biotechnol.* 15: 155-158.
